# P2X7 Receptor Antagonist Reduces Fibrosis and Inflammation in a Mouse Model of Alpha-Sarcoglycan Muscular Dystrophy

**DOI:** 10.3390/ph15010089

**Published:** 2022-01-13

**Authors:** Lizzia Raffaghello, Elisa Principi, Serena Baratto, Chiara Panicucci, Sara Pintus, Francesca Antonini, Genny Del Zotto, Andrea Benzi, Santina Bruzzone, Paolo Scudieri, Carlo Minetti, Elisabetta Gazzerro, Claudio Bruno

**Affiliations:** 1 Center of Translational and Experimental Myology, IRCCS Istituto Giannina Gaslini, 16147 Genoa, Italy; lizziaraffaghello@gaslini.org (L.R.); elisaprincipi@gaslini.org (E.P.); serenabaratto@gaslini.org (S.B.); chiarapanicucci@gaslini.org (C.P.); sarapintus@gaslini.org (S.P.); 2 Core Facilities, Department of Research and Diagnostics, IRCCS Istituto Giannina Gaslini, 16147 Genoa, Italy; francescaantonini@gaslini.org (F.A.); gennydelzotto@gaslini.org (G.D.Z.); 3 Section of Biochemistry, Department of Experimental Medicine (DIMES), University of Genoa, 16132 Genoa, Italy; andreeabenzi@gmail.com (A.B.); santinabruzzone@unige.it (S.B.); 4 Medical Genetic Unit, IRCCS Istituto Giannina Gaslini, 16147 Genoa, Italy; paoloscudieri@unige.it; 5 Department of Neuroscience, Rehabilitation, Ophtalmology, Genetics, Maternal and Child Health—DINOGMI, University of Genoa, 16147 Genoa, Italy; carlominetti@gaslini.org; 6 Pediatric Neurology Unit, IRCCS Istituto Giannina Gaslini, 16147 Genoa, Italy; 7 Unit of Muscle Research, Experimental and Clinical Research Center Charité Universitätsmedizin and Max Delbrück Research Center, 10627 Berlin, Germany

**Keywords:** muscular dystrophy, limb girdle muscular dystrophy, purinergic receptors

## Abstract

Limb-girdle muscular dystrophy R3, a rare genetic disorder affecting the limb proximal muscles, is caused by mutations in the α-sarcoglycan gene (Sgca) and aggravated by an immune-mediated damage, finely modulated by the extracellular (e)ATP/purinoceptors axis. Currently, no specific drugs are available. The aim of this study was to evaluate the therapeutic effectiveness of a selective P2X7 purinoreceptor antagonist, A438079. Sgca knockout mice were treated with A438079 every two days at 3 mg/Kg for 24 weeks. The P2X7 antagonist improved clinical parameters by ameliorating mice motor function and decreasing serum creatine kinase levels. Histological analysis of muscle morphology indicated a significant reduction of the percentage of central nuclei, of fiber size variability and of the extent of local fibrosis and inflammation. A cytometric characterization of the muscle inflammatory infiltrates showed that A438079 significantly decreased innate immune cells and upregulated the immunosuppressive regulatory T cell subpopulation. In α-sarcoglycan null mice, the selective P2X7 antagonist A438079 has been shown to be effective to counteract the progression of the dystrophic phenotype and to reduce the inflammatory response. P2X7 antagonism via selective inhibitors could be included in the immunosuppressant strategies aimed to dampen the basal immune-mediated damage and to favor a better engraftment of gene-cell therapies.

## 1. Introduction

Limb girdle muscular dystrophy R3 (LGMD R3), an autosomal recessive primary myopathy characterized by progressive involvement of the pelvic and shoulder girdles, is caused by mutations in the α-sarcoglycan gene (*SGCA*) [1,2]. *SGCA* encodes a transmembrane protein, α-sarcoglycan (α-SG), which, together with other 3 SG members (β, γ and δ), interacts with dystrophin, forming the dystrophin-glycoprotein complex (DGC) [3]. The DGC is crucially responsible for connecting the muscle fiber cytoskeleton to the extracellular matrix, preventing damage to the muscle fiber sarcolemma through shearing forces [4]. As other muscular dystrophies [5], LGMD R3 muscle histology is characterized by myofiber necrosis and regeneration, reactive fibrosis and adipose tissue substitution, reduced long-term regenerative capacity and inflammatory infiltrates [6,7,8]. In physiological conditions, skeletal muscle is considered a privileged immunological site characterized by few immune cells, poorly able to generate localized immune responses. In contrast, the dystrophic muscle presents a high level of inflammation associated with the activation of an innate and adaptive immune response [9,10]. In LGMD R3, the DGC disruption causes fragile muscle fibers and unstable sarcolemma, which, in turn, leads to muscle necrosis and to the release of damage-associated molecular pattern molecules (DAMPs), such as ATP, thus initiating a well-orchestrated immune reaction. Specifically, in α-SG-deficient muscle cells, the effect of ATP release can be further amplified since the ecto-ATPase activity of sarcoglycan, responsible for extracellular ATP (eATP) hydrolysis, is lost [11,12]. Once in the extracellular space, eATP binds and activates ionotropic (P2X) or metabotropic (P2Y) receptors [13]. Among P2X receptors, P2X7 has attracted vivid interest since it plays a relevant role in the induction of immune cell responses via inflammasome activation [14], and the consequent release of interleukin-1β (IL-1β) by mononuclear and polymorphonuclear phagocytes [14,15,16,17]. P2X7 is also over-expressed in dystrophic muscle cells [18,19,20,21], where it contributes to exacerbate myofiber injury by increasing sarcolemma permeability [22] and participates in the amplification of the inflammatory process by releasing IL-1β [20]. From these observations, P2X7 is an attractive therapeutic target, not only for reducing inflammation but also for decreasing myofiber damage and supporting the regenerative potential of dystrophic myoblasts [23]. In this respect, genetic ablation and pharmacological inhibition of the eATP-P2X7 axis by the broad-spectrum antagonist oxidized ATP (oATP) alleviated dystrophic phenotypes in mouse models of dystrophinopathy and sarcoglycanopathy [18,24,25]. In this study, we performed a long-term treatment in *Sgca null* mice with A438079, a potent and selective P2X7 antagonist. Functional, biochemical, cytofluorimetric and histological analysis are shown, providing evidence that A438079 improved muscle force and morphology by dampening the extent of muscle fibrosis and local inflammation.

## 2. Results

### 2.1. P2X7 Targeting by A438079 Improves Functional, Biochemical and Morphological Parameters in Sgca Mice

**Figure 1:**
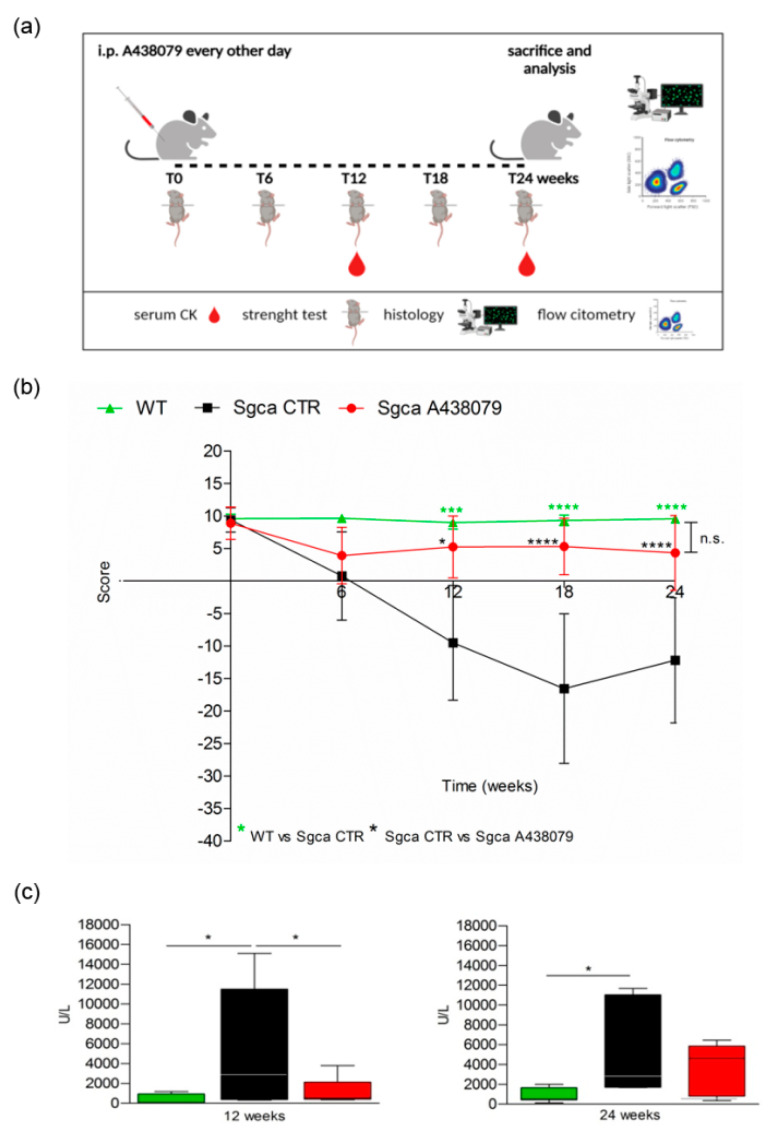
A438079 improves functional, biochemical and histological parameters in *Sgca* mice. (**a**): Experimental design: Four-week-old male *Sgca* mice were treated with PBS vehicle (*Sgca* CTR, *n* = 12) and A438079 (*Sgca* A438079 *n* = 8) that was administered intraperitoneally at the dose of 3 mg/Kg every other day for 24 weeks. Age-matched male C57BL/6 Wild Type (WT *n* = 12) mice were used as negative control; (**b**): four-limb hanging test was performed before treatment and at the end of 6, 12, 18 and 24 weeks of treatment. Each value represents the mean ± SD of animals evaluated. Statistical analysis was performed by one-way ANOVA followed by Tukey’s multiple comparison test. Green asterisks indicate statistical significance between WT and *Sgca* CTR (***, *p* < 0.001, ****, *p* < 0.0001). Black asterisks indicate statistical significance between *Sgca* CTR and *Sgca* A438079 (*, *p* < 0.05; ****, *p* < 0.0001). No statistical significance (n.s.) was identified between WT and *Sgca* A438079 mice; (**c**): serum creatine kinase (CK) levels were evaluated at the end of the twelfth and the twenty-fourth week of treatment. Blood samples were obtained by retro orbital withdraw from WT, *Sgca* CTR and *Sgca* A438079 mice. Data are expressed as mean ± SD of animals evaluated. Statistical analysis was performed by one-way ANOVA followed by Tukey’s multiple comparison test. Asterisks indicate statistical significance (*, *p* < 0.05).

In order to evaluate the therapeutic efficacy of a selective P2X7-targeting compound in an experimental model of α-sarcoglycanopathy, we treated four-week-old male *Sgca* knockout mice (also termed *Sgca-null*) mice with A438079, a specific antagonist of P2X7 [26]. The drug was administered to *Sgca-null* (here from referred as *Sgca*) mice by i.p. injections at the dose of 3 mg/kg every other day for 24 weeks (Figure 1a). *Sgca* mice injected with PBS (*Sgca* CTR) and Wild-Type (WT) mice served as controls.

The animals were weighted and followed once a week for signs of toxicity until the sacrifice. As shown in Supplementary Figure S1, the weight gain curve of *Sgca* mice treated with A438079 (*Sgca* A438079) was not significantly different from that of *Sgca* CTR mice. In addition, no signs of toxicity, including ruffled fur, vomiting, hyperactivity or loss of ambulation and breathing depression, were observed (data not shown). At the beginning (0 time) and after 6, 12, 18 and 24 weeks of treatment, animals were evaluated for muscle strength by the four-limb hanging test. Figure 1b shows that *Sgca* CTR mice progressively lost muscle strength up to 18 weeks (*Sgca* CTR vs. WT at 12 week *p* < 0.001, at 18 week *p* < 0.0001). At 24 weeks, they still showed significantly lower strength when compared to WT mice (*p* < 0.0001). The apparent slight recovery between 18 and 24 weeks was not significant. On the contrary, *Sgca* A438079 initially showed reduced functional performance, similarly to *Sgca* CTR mice, but after 6 weeks of treatment, muscle strength began to recover, almost reaching the performance of WT animals up to 24 weeks. The difference between *Sgca* CTR and *Sgca* A438079 mice at 12, 18 and 24 weeks was highly significant (*p* < 0.05 at 12, *p* < 0.0001 at 18 and 24 weeks). Accordingly, muscle strength in WT and *Sgca* A438079 mice was not significantly different at any time point. The efficacy of A438079 was confirmed by the analysis of serum levels of CK, a marker of muscle degeneration. As shown in Figure 1c, CK serum levels of *Sgca* CTR mice measured >10 times more than the WT levels (mean value of WT mice: 406 UI/l, *n* = 9; mean value of *Sgca* CTR mice: 5537 UI/l, *n* = 12, *p* < 0.05). Interestingly, A438079 treatment significantly reduced (by 76%) serum CK in *Sgca* mice after 12 weeks of treatment (mean value in *Sgca* A438079 mice: 1338 UI/l, *n* = 8 vs. *Sgca* CTR mice *p* < 0.05). After 24 weeks of treatment serum CK levels of *Sgca* CTR mice were 6.3 times higher than the WT levels and A438079 led to a serum CK decrease in the treated mice, although it did not reach statistical significance, likely due to increased variability of the measurements (mean value of WT mice: 829 UI/l, *n* = 12; mean value of *Sgca* CTR mice: 5235 UI/l, *n* = 10, *p* < 0.05).

To investigate whether the improved functional performance of *Sgca* A438079 mice correlated with decreased inflammation and muscle degeneration, we performed histological analysis by H&E staining of quadriceps from WT, *Sgca* CTR and *Sgca* A438079 mice.

**Figure 2:**
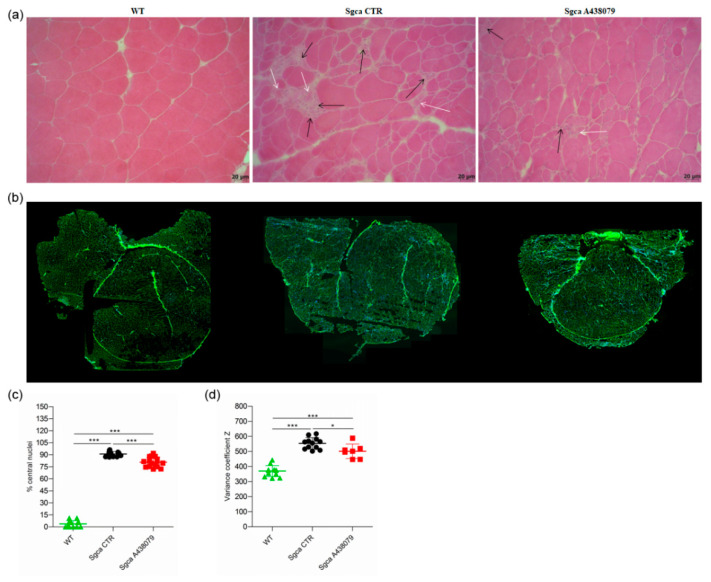
A438079 ameliorates the muscle morphology of *Sgca* mice. (**a**): frozen quadriceps tissue sections from WT, *Sgca* CTR and *Sgca* A438079 mice were collected at the end of the twenty-fourth week of treatment and stained with standard H&E technique. A representative image is shown. Black arrows indicate inflammatory infiltrates, white arrows indicate degenerating muscle fibers. Final magnification, 20×; (**b**): frozen quadriceps tissue sections from WT, *Sgca* CTR and *Sgca* A438079 mice were collected at the end of the twenty-fourth week of treatment and stained with wheat germ agglutinin (WGA) and DAPI. The whole quadricep section has been re-constructed; (**c**): percentage of central nuclei was quantified in four consecutive fields for each muscle section stained with wheat germ agglutinin (WGA) and DAPI and normalized for the fiber number of each field (WT *n*= 4; *Sgca* CTR *n* = 4; *Sgca* A438079 *n* = 4); (**d**): muscle fiber diameter variability from WT, *Sgca* CTR and *Sgca* A438079 mice was calculated in the whole area as variance coefficient Z of minimal Feret’s diameter (WT *n*= 12; *Sgca* CTR *n* = 12; *Sgca* A438079 *n* = 7). In panels (**c**,**d**), data are expressed as mean ± SD. Statistical analysis was performed by one-way ANOVA followed by Tukey’s multiple comparison test. Asterisks indicate statistical significance (*, *p* < 0.05; ***, *p* < 0.001).

Quadriceps from *Sgca* CTR mice presented areas of necrotic cells surrounded by reactive macrophage infiltration (Figure 2a) which were reduced upon A438079 treatment (Figure 2a). According to the histological analysis (Figure 2b), the percentage of centrally nucleated myofibers dramatically increased in quadriceps of *Sgca* mice in comparison to WT animals (*p* < 0.001) and was reduced by 12% in A438079-treated animals (*p* < 0.001) (Figure 2c). As expected, the fiber size variability, calculated as coefficient variance Z of minimal Feret’s diameter, was wider in *Sgca* CTR compared to WT mice (*p* < 0.001) but was significantly down-modulated by A438079 treatment (*p* < 0.05) (Figure 2d).

### 2.2. A438079 Reduces Muscular Fibrosis and Inflammation in Sgca Mice

**Figure 3:**
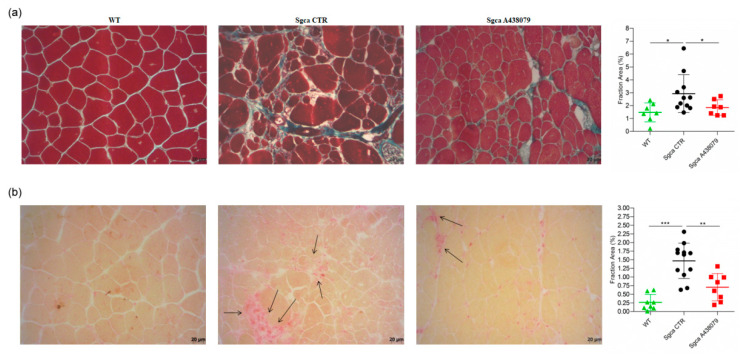
A438079 reduces muscle fibrosis and inflammatory infiltrates in *Sgca* mice. (**a**): frozen sections of quadriceps from WT (*n* = 7), *Sgca* CTR (*n* = 11) and *Sgca* A438079 (*n* = 7) were collected after 24 weeks and stained with a standard Masson trichrome stain protocol. A representative image is shown. Final magnification, 20×. In the right part of the panel (**a**) a graph of the fraction areas of fibrotic green positive signal/fraction area (%) of total section area of muscles evaluated is shown. Data are expressed as mean ± SD. Statistical analysis was performed by one-tailed ANOVA followed by Tukey’s multiple comparison test. Asterisks indicate statistical significance (*, *p* < 0.05); (**b**): frozen sections of quadriceps from WT (*n* = 8), *Sgca* CTR (*n* = 12) and *Sgca* A438079 (*n* = 8) were collected after 24 weeks and stained with an acid phosphatase technique. A representative image is shown. Black arrow indicates inflammatory infiltrates. Final magnification, 20×. In the right part of panel (**b**), a graph of the fraction areas of inflammatory red positive signal/fraction area (%) of total section area of muscles evaluated is shown. Data are expressed as mean ± SD. Statistical analysis was performed by one-way ANOVA followed by Tukey’s multiple comparison test. Asterisks indicate statistical significance (**, *p* < 0.01; ***, *p* < 0.001).

Fibrosis as characterized by replacement of muscle tissue with collagen deposits is the histopathological hallmark of end-stage muscular dystrophies, including alfa-sarcoglycanopathy [27]. In order to establish whether A438079 might impact collagen deposits, we performed a Masson trichrome staining on quadriceps of WT, *Sgca* CTR and *Sgca* A438079 mice and evaluated the fraction area of fibrotic reactions. As shown in Figure 3a, *Sgca* CTR quadriceps accumulated abundant extracellular matrix deposits which were increased in comparison to WT mice (mean value of WT mice: 1.47, *n* = 7; mean value of *Sgca* CTR mice: 2.92, *n* = 11, *p* < 0.05). A438079 treatment led to a 37% reduction of extracellular matrix deposition fraction area as compared to *Sgca* CTR mice (mean of *Sgca* A438079 mice: 1.85, *n* = 7, *p* < 0.05). No significant difference was observed between WT and *Sgca* A438079 animals.

To evaluate the anti-inflammatory effect of A438079, quadricep sections of WT, *Sgca* CTR and *Sgca* A438079 mice were stained with acid phosphatase, which provides a red positive signal in activated macrophages and degenerative myofibers. As shown in Figure 3b, the acid phosphatase-positive area fraction of quadriceps from *Sgca* CTR mice was increased in comparison to that of WT mice (mean value of WT mice: 0.26, *n* = 8; mean value of *Sgca* CTR mice: 1.47, *n* = 12, *p* < 0.001). Interestingly, A438079 led to 52% reduction of inflammatory area fraction of *Sgca* mice (mean of *Sgca* A438079 mice: 0.70, *n* = 8, *p* < 0.01). In contrast, no significant difference was observed between WT and *Sgca* A438079 animals.

### 2.3. A438079 Reduces Innate Inflammatory Cells and Increases T Regulatory Lymphocytes in Limb Muscles of Sgca Mice

**Figure 4:**
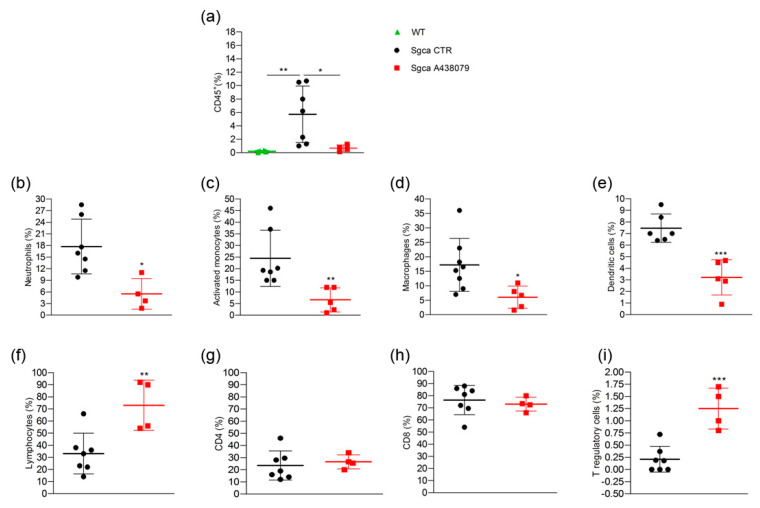
A438079 reduces innate inflammatory cells and increases T regulatory lymphocytes in muscles of *Sgca* mice. Flow cytometric analysis of immune cells isolated from a pool of gastrocnemius, quadriceps, anterior tibialis excised from WT (*n* = 6), *Sgca* CTR (*n* = 7) and *Sgca* A438079 (*n* = 5) mice and stained with specific anti-surface markers mAbs are shown; (**a**): percentage of CD45^+^ cells gated on alive cells; (**b**): percentage of Ly6G^+^/CD11b^+^neutrophils gated on CD45^+^ alive cells; (**c**): percentage of Ly6G^−^/Ly6C^+^/CD11b^+^ activated monocytes gated on CD45^+^ alive cells; (**d**): percentage of Ly6G^−^/F480^+^/CD11b^+^ macrophages gated on CD45^+^ alive cells; (**e**): percentage of Ly6G^−^/F480^−^/CD11c^+^ dendritic cells gated on CD45^+^ alive cells; (**f**): percentage of CD3^+^ T cells gated on CD45^+^ alive cells; (**g**): percentage of CD3^+^/CD4^+^ T cells gated on CD45^+^/CD3^+^ alive cells. (**h**): percentage of CD3^+^/CD8^+^ T cells gated on CD45^+^/CD3^+^ alive cells; (**i**): percentage of CD3^+^/CD4^+^CD25^+^/Foxp3^+^ T cells gated on CD3^+^ alive cells. Data are expressed as mean ± SD. Statistical analysis was performed by one-way ANOVA followed by Tukey’s multiple comparison test Panel (**a**) and unpaired *t*-test Panels (**b**–**i**). Asterisks indicate statistical significance (*, *p* < 0.05; **, *p* < 0.01; ***, *p* < 0.001).

In order to better characterize the phenotype of inflammatory muscle infiltrates, we performed a cytometric analysis of a pool of limb muscles, including gastrocnemius, quadriceps and anterior tibialis, isolated from WT, *Sgca* CTR and *Sgca* A438079 mice. As shown in Figure 4a, limb muscles of *Sgca* CTR mice were characterized by the presence of CD45^+^ hematopoietic immune cells which, in contrast, were not detected in WT mice (WT vs. *Sgca* CTR, *p* < 0.01).

A438079 treatment significantly reduced the percentage of CD45^+^ cells infiltrating the limb muscles of *Sgca* mice (*Sgca* CTR vs. *Sgca* A438079, *p* < 0.05). The further characterization of CD45^+^ cells was only performed for *Sgca* animals since the amount of CD45^+^ cells was negligible in the muscle of WT mice. *Sgca* A438079 mice presented a significant reduction of muscle infiltrating innate inflammatory cells, including Ly6G^+^/CD11b^+^ neutrophils (Figure 4b, *Sgca* CTR vs. *Sgca* A438079, *p* < 0.05), Ly6G^−^/CD11b^+^/Ly6C^+^ activated monocytes (Figure 4c, *Sgca* CTR vs. *Sgca* A438079, *p* < 0.01), Ly6G^−^/CD11b^+^/F480^+^ macrophages (Figure 4d, *Sgca* CTR vs. *Sgca* A438079, *p* < 0.05) and Ly6G^−^/CD11c^+^/F480^−^dendritic cells (DC) (Figure 4e, *Sgca* CTR vs. *Sgca* A438079, *p* < 0.001) in comparison to *Sgca* CTR animals. In contrast, the percentage of CD3^+^/CD4^+^/CD25^+^/Foxp3^+^ T regulatory (Treg) was significantly increased in the muscles of A438079 *Sgca* mice in comparison to *Sgca* CTR mice (Figure 4i, *Sgca* CTR vs. *Sgca* A438079, *p* < 0.001).

**Figure 5:**
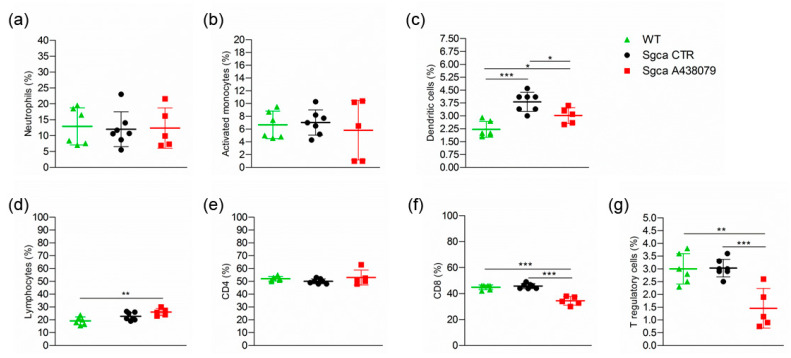
A438079 reduces peripheral cytotoxic and T regulatory cells in *Sgca* mice. Flow cytometric analysis of peripheral blood immune cells isolated from WT (*n* = 6), *Sgca* CTR (*n* = 7) and *Sgca* A438079 (*n* = 5) mice and stained with specific anti-surface markers mAbs are shown; (**a**): percentage of Ly6G^+^/CD11b^+^neutrophils gated on CD45^+^ alive cells; (**b**): percentage of Ly6G^−^/Ly6C^+^/CD11b^+^ activated monocytes gated on CD45^+^ alive cells; (**c**): percentage of Ly6G^−^/F480^−^/CD11c^+^ dendritic cells gated on CD45^+^ alive cells; (**d**): percentage of CD3^+^ T cells gated on CD45^+^ alive cells; (**e**): percentage of CD3^+^/CD4^+^ T cells gated on CD45^+^/CD3^+^ alive cells; (**f**): percentage of CD3^+^/CD8^+^ T cells gated on CD45^+^/CD3^+^ alive cells; (**g**): Percentage of CD3^+^ /CD4^+/^CD25^+^/Foxp3^+^ T cells gated on CD3^+^ alive cells. Data are expressed as mean ± SD. Statistical analysis was performed by one-way ANOVA followed by Tukey’s multiple comparison test. Asterisks indicate statistical significance (*, *p* < 0.05; **, *p* < 0.01; ***, *p* < 0.001).

Furthermore, the analysis of the peripheral blood (PB) immune cell populations of WT, *Sgca* CTR and *Sgca* A438079 mice showed that the dystrophic animals presented significantly higher percentages of DC compared to WT animals (Figure 5c, WT vs. *Sgca* CTR, *p* < 0.001 for DC). Treatment with A438079 significantly reduced the percentage of DC, CD8^+^ lymphocytes and T reg cells (Figure 5c,f,g, *Sgca* CTR vs. *Sgca* A438079, *p* < 0.05 for DC; *p* < 0.001 for CD8^+^ lymphocytes and *p* < 0.001 for Treg).

No significant changes were observed in the spleen of WT, *Sgca* CTR and *Sgca* A438079 mice, with the only exception of CD3^+^ T lymphocytes, which were slightly increased in *Sgca* A43879 mice vs. Sgca CTR (Supplementary Figure S2e, *Sgca* CTR vs. *Sgca* A438079, *p* < 0.01).

## 3. Discussion

In the present study, we provide evidence that the pharmacological inhibition of P2X7 by the selective antagonist A438079 attenuated the dystrophic phenotype of *Sgca* mice by reducing fibrosis and inflammation and improved muscle performance. P2X7 is an ATP receptor belonging to the ionotropic purinergic P2X subfamily, which is expressed on virtually all cell types of the immune system and regulates the innate and adaptive immune responses [28]. Alongside its expression on immune cells, P2X7 expression and function are upregulated in the dystrophic muscle [18,21,25,29,30,31]. In this context, we and other groups demonstrated that the genetic ablation of P2RX7 and its pharmacological inhibition by oATP, an irreversible, broad-spectrum P2X7 antagonist [17], produced significant improvements in key functional and molecular disease parameters in *mdx* and *Sgca* mice [18,24,25,30]. However, oATP can also interact with other P2X receptors, including P2X4 [32,33,34], and appears to exert anti-inflammatory effects, modulating the immune response independently of P2X7 blockage [33,35,36]. Therefore, experiments using oATP cannot unambiguously establish a role in inflammatory diseases for a specific member of the P2X family. Neurotoxicity has been described for oATP, likely due to the low specificity of the drug [37]. In order to overcome the above-described limitations, and to define the therapeutic effect of P2X7 targeting approaches in α-sarcoglycan-deficient muscular dystrophy, we specifically inhibited P2X7 using the A438079 molecule, one of the most potent and selective antagonists that competitively blocks P2X7 receptor in vitro activation and produces anti-nociceptive effects in in vivo settings [38,39,40]. In addition to nociception, A438079 has already been successfully used in in vivo models of hyperalgesia [41], epilepsy [42,43], Parkinson’s disease [44], salivary gland exocrinopathy [45], and Charcot-Marie-Tooth 1A disease [46]. Our results clearly show that, by targeting P2X7, A438079 ameliorated functional and morphological parameters in *Sgca* mice. In particular, A438079 improved muscle morphology by reducing the percentage of centralized nuclei and the coefficient variance Z of minimal Feret’s diameter (Figure 2c,d), which are typical signs of dystrophic damage [47]. Furthermore, according to previous studies [24], a relevant therapeutic effect exerted by A438079 was the reduction of muscle fibrosis (Figure 3a). P2X7 has been described to play a nodal role in triggering fibrosis through activation of multiple intracellular pathways that converge in inducing the collagen biosynthetic machinery in various organs [48]. As such, P2X7 blockade may interfere with the main pro-fibrotic pathways, thus possibly representing a target for the pharmacological modulation of fibrotic processes. The most evident beneficial effect of A438079 in *Sgca* animals was a significant modulation of muscle inflammation, a key feature of muscular dystrophies participating in the disease progression but also mediating muscle repair. The dichotomous role of inflammation has been extensively studied in Duchenne muscular dystrophy (DMD), in which CD4^+^ and CD8^+^ T cells, macrophages, eosinophils and natural killer T cells infiltrated both human and mouse dystrophic muscle [9,49]. In particular, proinflammatory monocytes CD11b^+^/Ly6G^−^/Ly6c^+^ have been reported to be the first innate immune cells to be mobilized from the bone marrow into the circulation and recruited to the site of tissue injury [50], such as dystrophic muscles, where they differentiated into inflammatory macrophages [51,52]. Different studies demonstrated that neutrophils actively participated in the exacerbation of muscular dystrophy and their specific depletion reduced muscular necrosis and inflammation in *mdx* mice [53,54,55]. Moreover, neutrophil-derived elastase impaired myoblast proliferation, survival and differentiation [56]. In line with this notion, we found that A438079 caused a significant reduction of innate immune cells, including neutrophils, activated monocytes and dendritic cells infiltrating the limb muscles of *Sgca* mice. The downregulation of innate immune response by P2X7 blockade was also observed in dystrophic mice (*mdx* and *Sgca*) treated with other P2X7 antagonists, i.e., oATP or zidovudine (AZT) [18,25,57]. However, the mechanism underlying the latter effect is still unclear. P2X7 antagonists could directly inhibit P2RX7 expressed by inflammatory innate immune cells infiltrating the dystrophic muscle. Alternatively, these agents might reduce inflammatory cell migration into the injured tissue. In favor of the first hypothesis, innate immune cells are known to express functional P2X7, which in turn triggers inflammasome activation [17,28,58,59,60]. The second hypothesis is sustained by data showing P2X7-dependent release of chemotactic factors by macrophages. These factors, including CXCL2/macrophage inflammatory protein-2 (MIP-2), were involved in the recruitment of neutrophils into the injured tissue [61]. The immune phenotype of limb muscle from *Sgca* mice treated with A438079 also showed that the pharmacological treatment significantly increased Foxp3^+^ Treg without affecting CD4^+^ and CD8^+^ T lymphocytes. These findings are consistent with previous studies in which P2X7 blockade by oATP or P2X7 genetic ablation in dystrophic mice resulted in a significant increase of Foxp3^+^ Treg [18,24,25]. Treg have been described to play a dual beneficial role in dystrophic muscles. On one side, they suppress type 1 inflammation by secreting IL-10; on the other side, Treg may also have direct effects on muscle growth and regeneration through secretion of amphiregulin, an epidermal growth factor family member whose receptors are expressed on muscle satellite cells that are critical for muscle regeneration [62,63]. Interestingly, our results showed that A438079 exerted not only a local but also a systemic anti-inflammatory effect by reducing circulating CD8^+^ cytotoxic T lymphocytes in *Sgca* mice. Currently, physical therapy and prevention of secondary cardiac, pulmonary or orthopedic complications are the only possible care interventions. Although a chronic inflammatory response is documented in muscle specimens from LGMD R3 patients, no trials assessing the effects of immunosuppressive therapies have been proposed in alpha-sarcoglycan deficiency. However, two unrelated LGMD R3 patients treated with steroids showed clinical improvement [64,65,66]. In light of these considerations, strategies aimed at reducing muscle inflammation, increasing the amount of Treg infiltrating an injured muscle and exerting a systemic anti-inflammatory effect, i.e., antagonists of P2X7, might represent a therapeutic approach for LGMD R3. Immunomodulatory regimens become even more relevant as new gene therapies or gene editing approaches are being developed for alpha-sarcoglycanopathy [67,68,69], with gene therapy being in Phase I/II clinical trials for LGMD R3 patients (NCT01976091; NCT00494195). Since the success of gene therapy in the muscle tissue has to challenge the pre-existing status of chronic tissue inflammation typically identified in this diseases [67,68,69,70,71], our data suggest that P2X7 antagonism might represent a good strategy to dampen chronic inflammation, possibly leading to a better delivery of gene therapy. To date P2X7 selective antagonists have already been tested in Phase I/II clinical trials for the treatment of Crohn’s disease, rheumatoid arthritis, basal cell carcinoma with an overall good tolerability and variable efficacy [72] and a new trial is currently ongoing assessing the effects of JNJ-54175446, a potent, brain-penetrant, selective P2X7 antagonist [73] in patients with major depressive disorder (ClinicalTrials.gov Identifier: NCT04116606).

## 4. Materials and Methods

### 4.1. In Vivo Experiments

C57BL/6 wild-type (WT) and *Sgca* knockout mice (also termed *Sgca-null*) were bred in the Animal Facility at Policlinico San Martino, Genova. All mice were housed under standard specific pathogen–free conditions and allowed access to food and water ad libitum. All experimental protocols were approved by the Policlinico San Martino Animal Welfare Body and by the Italian Ministry of Health (Authorization n° 215–2018-PR). *Sgca* mice were previously described [74]. The study design defined a minimum goal of a *n* = 8 animals per genotype and experimental group. According amounts of the drug A438079 was acquired from Tocris Bioscience Bristol, UK. Four week old *Sgca* male mice were randomly divided into two groups: one treated by i.p. injections with A438079 at 3 mg/Kg every other day in the morning between 10–12 for 24 weeks (*Sgca* A438079) and one treated with the same volume of phosphatase-buffered saline (PBS, Sigma Aldrich, St. Louis, MO, USA) every other day for 24 weeks (*Sgca* Control: Sgca CTR). In consideration of the high variability of the histological markers in mouse models of muscular dystrophies [18,25], and in order to increase the statistical power of the study a n of 12 WT mice and a n of 12 *Sgca* CTR were included. The dose of 3 mg/kg of A438079 proved to be well tolerated by wild-type rats [46]. A438079 was reconstituted at a final concentration of 1 mg/mL in PBS and stored at −20 °C; the reconstituted drug was thawed and immediately used at a final concentration of 3 mg/Kg. All animals were euthanized at the end of treatment by carbon dioxide inhalation and muscles were collected for histological and cytofluorimetric analysis. A group of age-matched WT C57Bl/6 male mice was used as internal control. All animals were weighed and followed for any sign of toxicity, including ruffled fur, vomiting, hyperactivity or loss of deambulation and breathing depression once a week. Blood samples from *Sgca* CTR and *Sgca* A438079 mice were obtained from the saphenous vein (90 µL) before treatment and 12 and 24 weeks after treatment. The samples were centrifuged at 3600× *g* for 30 min and immediately after centrifugation the serum was isolated and stored at −80 °C. Serum creatine kinase (CK) levels were measured using a clinical-standard automatic chemistry analyzer (BS-380 Mindray, Milan, Italy).

### 4.2. Four-Limb Hanging Test

Before treatment and at the end of the sixth, twelfth, eighteenth and twenty-fourth week of treatment, the muscle strength of WT, *Sgca* CTR and *Sgca* A438079- mice was scored through the four-limb hanging test. Mice were subjected to a 180-s lasting hanging test, during which a falling score was recorded. The animals had to hang for three trials, and the average maximum hanging time of the three trials was measured (standard operating procedure, https://treat-nmd.org/research-overview/preclinical-research/experimental-protocols-for-dmd-animal-models, last accessed on September 2021.

### 4.3. Histological Studies, Imaging and Analysis

Quadriceps isolated from WT, *Sgca* CTR and *Sgca* A438079 mice were cut on cryostat, and 7-μm-thick sections were stained with standard hematoxylin and eosin (H&E) (reagents from Sigma Aldrich), acid phosphatase (reagents from Sigma Aldrich) to detect inflammatory reactions and Masson trichrome (reagents from Sigma Aldrich) to evaluate muscle fibrosis. Representative pictures were taken at 20× magnification. To quantify the extension of the inflammatory response and fibrotic area, images of stained sections were acquired using a Nikon Ti Eclipse microscope equipped with a 20× objective. Whole sections were imaged with an automated tile scan acquisition (usually over a 45 mm^2^-surface) by using the perfect focus system (PFS) to control the focal plane. Quantification of acid phosphatase and trichrome staining was performed using semi-automated measurement tools in NIS-Elements AR software version 4.20 and expressed in terms of fraction area (the ratio between total section area and the area of the stained objects that were detected by HSI thresholding mode). All the histological analyses were performed blind to experimental group identity. The Masson Trichrome and acid phosphatase staining were completely negative in WT mice; therefore, the n was reduced to 7 animals.The histological sections of *Sgca* CTR and *Sgca* A438079 treated mice displaying freezing artifacts were not analyzed and are excluded from the results.

### 4.4. Immunofluorescence

The wheat germ agglutinin (WGA)/DAPI (WGA Alexa Fluor™ 488 Conjugate Invitrogen, Thermo Fisher Scientific, Waltham, MA, USA; DAPI, Fluoromount-G^®^ Southern Biotech, Birmingham, USA) staining was performed on quadricep 5-μm-thick sections, in order to calculate the Minimum Feret’s Diameter and the percentage of centralized nuclei. Briefly, unfixed quadriceps sections were incubated with a blocking solution containing 0.2% TritonX-100 (Sigma Aldrich), 2% bovine serum albumin (Sigma Aldrich), 5% fetal bovine serum (GIBCO, Thermo Fisher Scientific), 2% goat serum (GIBCO) in PBS for 1 h at room temperature (RT) and then with WGA Alexa Fluor 488 conjugated diluted 1:200 in Hank’s Balanced Salt Solution (HBSS) (GIBCO) for 2 h at RT. Finally, sections were mounted with Fluoromount G. Images were acquired by Axioplan Imager M2 miscroscope software AxioVs40 version 4.8.2.0 (Zeiss, Oberkochen, Germany) and manually overlapped using Adobe Photoshop CS6 to generate whole cross-section. Image analysis was performed using Fiji, ImageJ 1.52i (NIH, Bethesda, MA, USA). A plugin (Muscle Morphometry) developed as described in [24] was used to quantify the muscle fiber diameter (minimal Feret’s diameter) and the percentage of centralized nuclei, as described in TREAT-NMD-recommended protocol (https://treat-nmd.org/research-overview/preclinical-research/experimental-protocols-for-dmd-animal-models, last accessed on 10 September 2021). All the analyses were performed blind to experimental group identity.

### 4.5. Flow Cytometry

Hematopoietic cells were collected from different districts, namely PB, spleen and limb muscles. Cells collected from PB were first incubated with 2 μL/sample of TruStainFcX^TM^ anti-mouse CD16/32 for 5 min in order to block Fc receptor and then with cocktails of antibodies specific for CD45, CD3, CD4, CD8, CD11b, CD11c, CD25, F4/80, Ly-6C, Ly-6G and Foxp3 for 30 min. The intracellular staining of transcription factor Foxp3 was performed using the Foxp3/Transcription factor staining buffer set (Thermo Fisher) as described by the manufacturer. After antibody incubation, samples were lysed (Becton Dickinson Pharm Lyse TM, San Josè, CA, USA), washed and resuspended in 300 ul of PBS. All the antibodies were purchased from Biolegend (San Diego, CA, USA). Gastrocnemius, quadriceps and anterior tibialis excised from WT, *Sgca* CTR and *Sgca* A438079 mice were resuspended in RPMI 1640 base medium (Euro Clone, Milan, Italy), mechanically and enzimatically digested using Skeletal Muscle Dissociation Kit (Miltenyi Biotec, Bologna, Italy) and filtered through 100- and 70-μm mesh filters (BD Bioscience, San Jose, CA, USA) (Supplementary Method). After filtration, cells were purified using gradient centrifugation by Percoll solution (GE Healthcare Bio-sciences, Uppsala, Sweden) and stained with Live/Dead^TM^ Fixable Yellow Dead Cell Stain Kit (Invitrogen, Thermo Fisher Scientific) and the antibodies listed above. The spleen from WT, *Sgca* CTR and *Sgca* A438079 mice was mechanically digested, filtered through 100- and 70-μm mesh filters, counted and stained as described for PB and muscle. All acquisitions were performed with a three laser LSR Fortessa X20 (Becton Dickinson) and obtained FSC files were analysed with Kaluza Software (version 2.1, Beckman Coulter). The immune profile of the peripheral blood, spleen and muscles was performed in the same animals (WT *n* = 6, *Sgca* CTR *n* = 7 and *Sgca* A438079 *n* = 5).

### 4.6. Statistical Analysis

Statistical parameters, including the exact value of *n* and statistical significance, are reported in the figures and their associated legends. Results were analyzed using a one-way ANOVA followed by Tukey’s multiple comparison test or unpaired *t*-test, where indicated, using GraphPad Prism 3.0 software (GraphPad Software, El Camino Real, San Diego, CA, USA). Asterisks indicate statistical significance (*, *p* < 0.05; **, *p* < 0.01; ***, *p* < 0.001, ****, *p* < 0.0001).

## 5. Conclusions

In conclusion, A438079 ameliorated the dystrophic phenotype of *Sgca* mice by reducing muscle fibrosis and inflammation and by improving functional muscle performance. In the current scenario of clinical trials including gene therapy, selective P2X7 antagonists could represent candidates for a combinatory therapy to endorse the efficacy of disease-specific gene therapy by dampening the basal muscular inflammation.

## Data Availability

The data presented in this study are available in the main text and Supplementary Material.

## Supplementary Materials

The following are available at https://www.mdpi.com/article/10.3390/ph15010089/s1. Figure S1: Weight of *Sgca* mice treated (not) with A438079; Figure S2: A438079 modulation of splenic immune cells of *Sgca* mice and Supplementary Methods.
