# Supplementary material for: P2X7 Receptor Antagonist Reduces Fibrosis and Inflammation in a Mouse Model of Alpha-Sarcoglycan Muscular Dystrophy
Source: Pharmaceuticals (Basel). 2022 Jan 13;15(1):89. doi: 10.3390/ph15010089 (PMC8777980; doi:10.3390/ph15010089)
Supplement: Supplementary file 1 [file pharmaceuticals-15-00089-s001.zip › Supplementary method.pdf]

Supplementary method

Cytofluorimetric analysis

The isolation of inflammatory cells from the muscle tissue for the cytofluorimetric analysis was completed through two methods:

- Method A: enzymatic digestion with Liberase TL at 0.25 mg/mL (Roche, Milan, Italy) and DNAse at 0.5 mg/mL (Roche) for 3 hours followed by overnight incubation with RPMI + 10% Fetal Bovine Serum (FBS) (Euro Clone, Milan, Italy), filtration through 100 and 70 µm mesh filters (BD Bioscience, San Jose, CA, USA) and cytometric analysis (modified from Liang et al. J Imm Methods 2015) .
- Method B: enzimatic digestion using Skeletal Muscle Dissociation Kit (Miltenyi Biotec, Bologna, Italy) and filtration through 100 and 70 µm mesh filters, purification using gradient centrifugation by Percoll solution (GE Healthcare Bio-sciences, Uppsala, Sweden) and analysis.

  The high number of dead cells resulting from Method A did not allow a reliable analysis of immune cell populations, while Method B proved efficient in excluding dead cells, thus providing clean results. In the manuscript we describe the mice which were analysed with Method B (WT (n=6), *Sgca* CTR (n=7) and *Sgca* A438079 (n=5).
