# Supplementary figures and images for: P2X7 Receptor Antagonist Reduces Fibrosis and Inflammation in a Mouse Model of Alpha-Sarcoglycan Muscular Dystrophy
Source: Pharmaceuticals (Basel). 2022 Jan 13;15(1):89. doi: 10.3390/ph15010089 (PMC8777980; doi:10.3390/ph15010089)

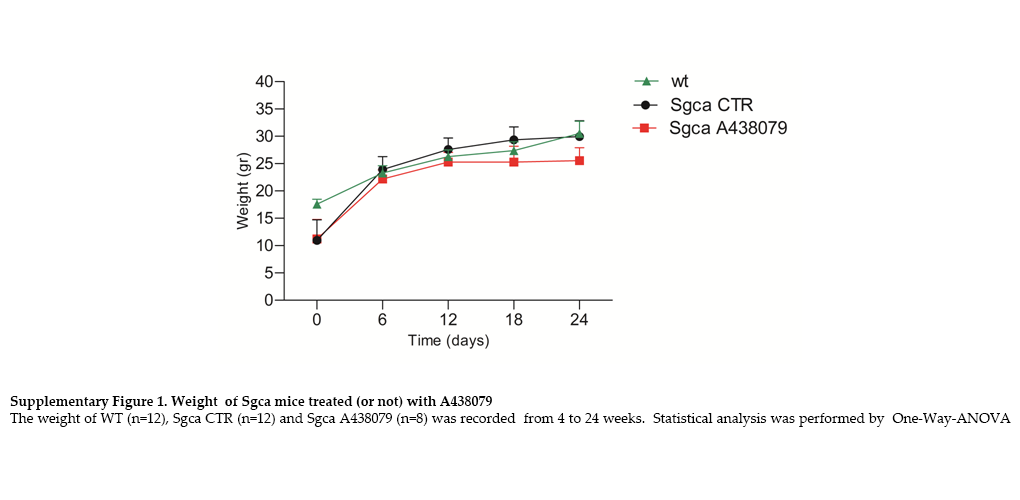

Supplement: Supplementary file 1 [file pharmaceuticals-15-00089-s001.zip › supplementary 1 REVISED DEF.tif]

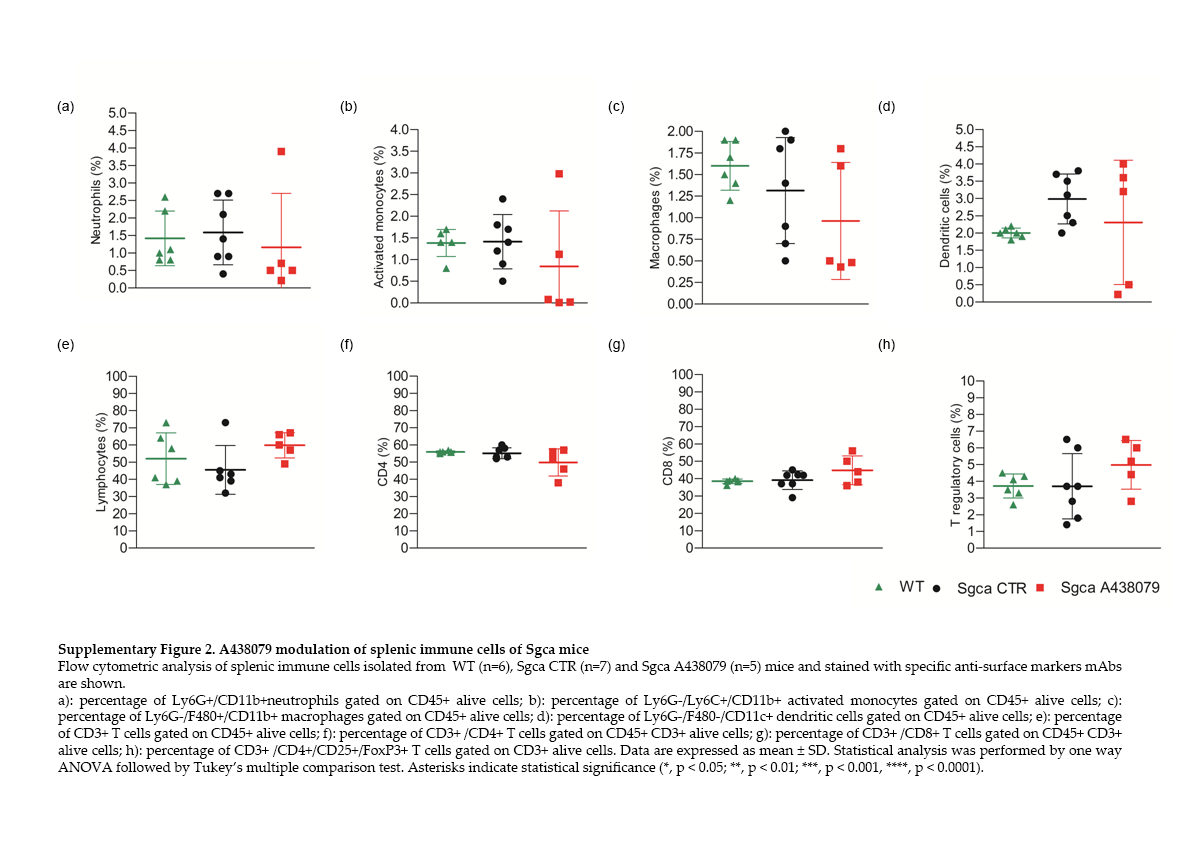

Supplement: Supplementary file 1 [file pharmaceuticals-15-00089-s001.zip › supplementary 2 REVISED DEF.tif]
